# Expression of toll-like receptors in non-endemic nasopharyngeal carcinoma

**DOI:** 10.1186/s12885-019-5816-9

**Published:** 2019-06-25

**Authors:** Miia Ruuskanen, Ilmo Leivo, Heikki Minn, Tero Vahlberg, Caj Haglund, Jaana Hagström, Heikki Irjala

**Affiliations:** 1 Department of Otorhinolaryngology - Head and Neck Surgery, Turku University Hospital and University of Turku, Kiinamyllynkatu 4-8, 20521 Turku, Finland; 2 Department of Pathology, University of Turku and Turku University Hospital, Kiinamyllynkatu 4-8, 20521 Turku, Finland; 3 Department of Oncology, University of Turku and Turku University Hospital, Kiinamyllynkatu 4-8, 20521 Turku, Finland; 4 Department of Biostatistics, Turku University Hospital and University of Turku, Kiinamyllynkatu 4-8, 20521 Turku, Finland; 5 Department of Surgery, University of Helsinki and Helsinki University Hospital, Haartmaninkatu 4, 00029 HUS Helsinki, Finland; 6 Research Programs Unit - Translational Cancer Biology Program, University of Helsinki, Haartmaninkatu 3 C, 00029 HUS Helsinki, Finland; 7 Department of Pathology, University of Helsinki and Helsinki University Hospital, Haartmaninkatu 3 C, 00029 HUS Helsinki, Finland

**Keywords:** Nasopharyngeal carcinoma, Head and neck cancer, Toll-like receptor, Epstein-Barr virus, Human papillomavirus

## Abstract

**Background:**

Nasopharyngeal carcinoma (NPC) is a malignant disease with an enigmatic etiology. NPC associates with Epstein-Barr virus (EBV) and human papillomaviruses (HPVs), while immunological factors also play a role in carcinogenesis. Toll-like receptors (TLRs) are pattern recognition receptors that participate in the immunological defence against pathogens, but their functions are also linked to cancer.

**Methods:**

In our whole population-based study, we retrieved 150 Finnish NPC cases and studied their tumour samples for TLR1, TLR2, TLR4, TLR5, TLR7, and TLR9 expressions by immunohistochemistry, and for the presence of EBV and high-risk HPVs with EBV RNA and HPV E6/E7 mRNA in situ hybridizations. In addition, we analyzed the TLR expression patterns according to age, tumour histology, EBV/HPV status, and outcome.

**Results:**

We found that all TLRs studied were highly expressed in NPC. Viral status of the tumours varied, and 62% of them were EBV-positive, 14% HPV-positive, and 24% virus-negative. The tumours with strong TLR2^nucl^ or TLR5 expression were mostly virus-negative or HPV-positive keratinizing squamous cell carcinomas, and the patients with these tumours were significantly older than those with mild or negative TLR2^nucl^/TLR5 expression. In Kaplan-Meier analysis, the patients with strong TLR5 expression had worse survival compared to the patients with negative or mild TLR5 expression, but the results were linked to other patient and tumour characteristics. In multivariable-adjusted Cox regression analysis, the patients with positive TLR7 tumour expression had better overall survival than those with no TLR7 expression. The 5-year overall survival rates according to TLR7 expression were 66% (mild), 52% (moderate or strong), and 22% (negative).

**Conclusions:**

TLRs are highly expressed in non-endemic NPC. Intensity of TLR2 and TLR5 expressions correlate with viral status, and TLR7 seems to be an independent prognostic factor of non-endemic NPC.

## Background

Toll-like receptors (TLRs) are a family of transmembrane receptors that play an important role in innate immune defence. TLRs recognize the receptor-specific pathogen-associated molecular patterns (PAMPs) of microbes, and respond by activating immune cells against them [1]. TLRs also recognize the endogenous damage-associated molecular patterns (DAMPs) released from injured tissues, and TLR pathways have been shown to maintain tissue homeostasis by regulating wound healing processes and apoptosis [2–4]. In humans, ten different TLRs (TLR1-10) have been characterized [1]. For TLR1-9, several specific ligands have been identified, whereas the ligand for TLR10 remains elusive [1, 5]. TLR1, TLR2, TLR4, TLR5, and TLR6 were originally characterized as exclusively expressed on the cell surface and TLR3, TLR7, TLR8, and TLR9 as almost exclusively expressed in intracellular compartments such as endosomes [1]. The subcellular localization of TLR10 has not yet been characterized [5]. Nevertheless, recent findings suggest that TLR localization may be altered across cell types and in response to stimulation or disease [5]. Many studies have shown changes in TLR expression with oncogenic transformation [6]. However, the actual function of cancer-associated TLR modulation remains controversial. TLR stimulation may have anti- or pro-tumoral effects depending on the TLR receptor and cancer type [6]. Over the past decade, interest in the role of TLRs in tumorigenesis has increased, and numerous preclinical and clinical trials are ongoing to develop TLR agonists for cancer therapy [7].

Nasopharyngeal carcinoma (NPC) is a highly invasive malignant tumour arising from the mucosal epithelium of the nasopharynx. NPC has marked geographical disparities in incidence, with the highest occurrence in Southeast Asia and lowest in Europe and North America [8]. NPC is subdivided into three major histological types: keratinizing squamous cell carcinoma (KSCC), non-keratinizing carcinoma, and basaloid squamous cell carcinoma [9]. Non-keratinizing carcinoma can be further subdivided into undifferentiated and differentiated types [9]. The etiology of NPC is poorly understood, but epidemiological studies indicate that both genetic and environmental factors contribute to its development [10]. In high-incidence endemic areas, more than 95% of NPC tumours show non-keratinizing histology with Epstein-Barr virus (EBV) association, while in low-incidence non-endemic areas, the histology and viral status are more diverse. In the latter areas, KSCCs and human papillomavirus (HPV)-positive tumours are additionally found [11–13]. Although it is commonly accepted that EBV and HPV can contribute to carcinogenesis, these viruses alone seem insufficient for malignant transformation [14–16]. Recent studies indicate that the host’s immunological responses to viruses and precancerous lesions have an important role in cancer development, and that tumour progression could partly be due to a failure in the innate immune response [16]. In HPV-positive oropharyngeal squamous cell carcinoma (OPSCC), strong expression of TLR5 and low expression of TLR7 has been associated with poor disease-specific survival [17]. Furthermore, the intensity of TLR4 expression has been significantly lower in laryngeal papillomas transforming into laryngeal squamous cell carcinoma than in papillomas without malignant transformation [18]. In NPC, the importance of TLRs in tumour immunity has been demonstrated in studies from endemic areas, where certain sequence variants in TLR genes were associated with increased NPC risk [19–22]. However, data on TLR expression in NPC is scarce and limited to endemic cases.

The purpose of this whole population-based study on NPC was to characterize the expression of six TLRs of interest for malignant transformation and immune activation. We studied the expression of TLR1, TLR2, TLR4, TLR5, TLR7, and TLR9 in Finnish patients and related the findings to histopathological subtypes, viral status, and survival.

## Methods

### Patients

We identified a total of 207 patients with primary NPC, which were diagnosed between 1990 and 2009, from the files of the nationwide Finnish Cancer Registry [23]. Clinical records for patient and disease characteristics, treatment and follow-up data were collected from eight major hospitals in Finland. All tumours were confirmed to originate from the nasopharyngeal epithelium. The clinical stage was determined according to the International Union Against Cancer (UICC) TNM staging system, 7th edition [24], and the dates and causes of death were acquired from the Finnish Cancer Registry and Statistics Finland. The study was approved by the Research Ethics Committee of the Hospital District of Southwest Finland, National Institute for Health and Welfare, and National Supervisory Authority for Welfare and Health.

### Tissue microarray construction

Histological slides from NPC biopsies, taken at the time of diagnosis and stained with hematoxylin and eosin, were available for 168 patients. Tumour slides were reviewed and reclassified by an experienced head and neck pathologist (I.L.) according to the 4th edition of the World Health Organization (WHO) histological classification [9]. Formalin-fixed paraffin-embedded (FFPE) tissue blocks were available from 150 patients. Tissue microarrays (TMAs) were constructed using an automated tissue microarrayer (TMA Grand Master, 3D Histech Ltd., Budapest, Hungary) to create five new paraffin blocks from 1 mm core samples (*n* = 324) containing representative areas of NPC tumour tissue taken from the original FFPE blocks of tumours. Each patient with an available tumour block was represented in the array by at least one core, usually two. Scores from the duplicate cores were averaged to produce a single score. Five of the original tumour blocks represented neck metastases, while the remaining 145 were from the primary tumours. The TMAs also included as control tissues cores from normal liver and placenta. In addition, five samples from benign hyperplastic adenoid were stained separately for benign controls. In the final TMAs, representative tumour samples were lacking due to technical reasons in one patient for TLR1, in nine patients for TLR2 and TLR4, in seven patients for TLR5 and TLR7, and in one patient for TLR9.

### Immunohistochemistry for TLRs

Immunohistochemical stains were performed on 3.5-μm thick TMA sections, which were mounted on glass slides, deparaffinized in xylene, and rehydrated in graded alcohol series. The sections were treated in a PreTreatment module (Lab Vision Corp, UK Ltd., Altrincham, UK) in Tris-HCl buffer (pH 8.5; TLR2, TLR4, and TLR7) or in Tris-EDTA buffer (pH 9.0; TLR1, TLR5, and TLR9) at 98 °C for 20 min. Endogenous peroxidase activity was blocked with 0.3% Dako REAL Peroxidase-Blocking Solution (Dako, Glostrup, Denmark) for 5 min. Immunostaining was performed in an Autostainer 480 (LabVision Corp, Fremont, CA, USA) incubating sections with primary antibodies against TLRs (Table 1) for 60 min (TLR9) or overnight (TLR1, TLR2, TLR4, TLR5, TLR7) followed by a 30 min incubation with Dako REAL EnVision Detection System (peroxidase/DAB+, rabbit/mouse). Between each step, slides were washed with PBS-0.04%-Tween20. Sections were counterstained with Dako Meyer’s hematoxylin and mounted in PERTEX (Histolab Product AB, Göteborg, Sweden). For each TLR, the five tumour TMAs were stained simultaneously in one and the same staining procedure, and five benign hyperplastic adenoid tissues were also stained for staining control comparison. Furthermore, the authors are experienced by previous use of these antibodies in various other tumour materials [17, 18].

**Table 1:** TLR antibodies used and TLR staining patterns in NPC and normal controls

Antigen	Primary antibody	Dilution	Staining pattern in NPC	Staining pattern in normal nasopharyngeal epithelial cells
TLR1	sc-30,000, polyclonal, rabbit, Santa Cruz Biotechnology Inc., CA, USA	1:100	Cytoplasmic	Cytoplasmic
TLR2	sc-10,739, polyclonal, rabbit, Santa Cruz Biotechnology Inc., CA, USA	1:200	Nuclear and cytoplasmic	Nuclear and cytoplasmic
TLR4	sc-10,741, polyclonal, rabbit, Santa Cruz Biotechnology Inc., CA, USA	1:300	Cytoplasmic	Nuclear and cytoplasmic
TLR5	NBP2–24787, monoclonal, mouse, Novus Biologicals, CO, USA	1:100	Nuclear membranous and cytoplasmic	Nuclear membranous and cytoplasmic
TLR7	IMG-581A, polyclonal, rabbit, Imgenex/Novus Biologicals, CO, USA	1:300	Nuclear membranous and nuclear	Nuclear and cytoplasmic
TLR9	sc-25,468, polyclonal, rabbit, Santa Cruz Biotechnology Inc., CA, USA	1:100	Cytoplasmic	Plasma membranous and cytoplasmic

### Evaluation of TLR immunoreactivity in NPC

**Fig. 1:**
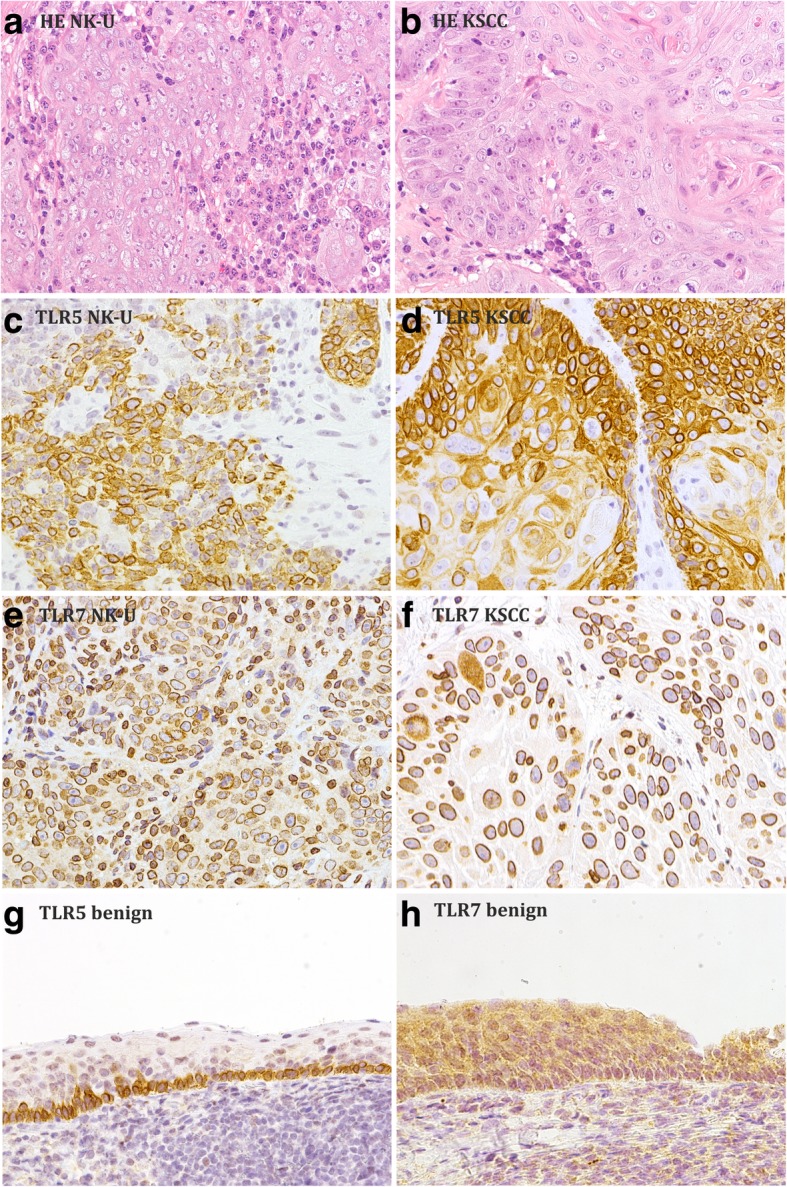
TLR5 and TLR7 expression in nasopharyngeal carcinoma and in the normal epithelium. **a** H&E staining in non-keratinizing undifferentiated carcinoma (NK-U). **b** H&E staining in keratinizing squamous cell carcinoma (KSCC). **c** Mild positive TLR5 expression in NK-U. **d** Strong positive TLR5 expression in KSCC. **e** Moderate positive TLR7 expression in NK-U. **f** Moderate positive TLR7 expression in KSCC. **g** Positive TLR5 staining in benign nasopharyngeal epithelium. **h** Positive TLR7 staining in benign nasopharyngeal epithelium. Magnification × 250

The interpretation of immunopositivity was performed by two head and neck pathologist (I.L. and J.H.), who were blinded to the clinical data (Fig. 1 and Table 1). TLR positivity was scored using a semi-quantitative scoring method where classification into negative, mild, and strong staining was based on the intensity of staining in cytoplasmic and nuclear areas. For a positive scoring, it was required that all or a large majority (> 80%) of tumour cells in the sample stained positively. Staining for all studied TLRs was scored in malignant NPC tissues. The scoring method has been documented previously [17], and representative examples are illustrated in Fig. 1. Using the scoring method, TLR1 positivity was classified as mild or strong granular cytoplasmic signals. TLR2 was expressed in both the cytoplasm and the nuclei, and the intensity of nuclear expression varied independently of cytoplasmic expression. Thus, cytoplasmic (TLR2^cyto^) and nuclear (TLR2^nucl^) positivities were scored separately as mild or strong. Nuclear expression was scored mild when the intensity of the staining was weaker or similar to cytoplasmic intensity, and strong when the staining was more intensive in the nuclei than in the cytoplasm. TLR4 positivity was scored mild or strong based on the intensity of cytoplasmic staining. TLR5 was expressed in the cytoplasm and on the nuclear membranes, and the two staining intensities were directly related: if cytoplasmic staining was strong, there was also strong staining on the nuclear membranes. Thus, the two were scored together as one TLR5 score: negative, mild (Fig. 1c) or strong (Fig. 1d). TLR7 staining was scored mild if some nuclear membranes were positive, moderate if all nuclear membranes and some nuclei were positive (Fig. 1e, f), and strong if nuclear membranes and nuclei stained substantially. TLR9 positivity was scored by the intensity of cytoplasmic staining as mild or strong.

### In situ hybridization for EBV-encoded RNA and HPV E6/E7 mRNA

In situ hybridization (ISH) for EBV-encoded RNA (EBER) and for high risk HPV E6/E7 mRNA were performed on 3.5-μm thick TMA sections as described earlier [13]. This high risk HPV cocktail probe (RNAscope, Advanced Cell Diagnostics, Inc., Hayward, CA, USA) detects HPV genotypes 16, 18, 26, 31, 33, 35, 39, 45, 51, 52, 53, 56, 58, 59, 66, 68, 73 and 82. Positive hybridization for EBER was defined as strong diffuse signals in the nucleus of nearly all (> 90%) tumour cells. A positive HPV E6/E7 mRNA staining was defined as punctate staining that co-localised to the cytoplasm and/or nucleus of any of the malignant cells. The positivity was graded mild when at least 10 cells were intensively positive or less than 50% of the cells were positive with low intensity, moderate when over 50 to 70% of the tumour cells were positive, and strong when strong intensity of dot-like nuclear and cytoplasmic signals were present in over 70% of the cells or nearly all tumour cells were positive with low intensity.

### Immunohistochemistry for p16

Immunohistochemical stains were performed on 3.5-μm thick TMA sections as described earlier, and p16 expression was defined positive when there were strong diffuse nuclear and cytoplasmic staining in 75% or more of tumour cells [13].

### Treatment

Patients were treated with radiotherapy with or without concurrent chemotherapy as described earlier [13]. Typically patients received 2 Gy daily fractions five times per week, and the median dose was 70 Gy in the nasopharyngeal tumour area, 60 Gy in the involved lymph nodes, and 50 Gy in the elective neck area. The median treatment time was 7 weeks. In total, 76 patients (51%) received concurrent chemotherapy usually (87%) with platinum based cytostatic drugs. Seven patients (4%) with a compromised general condition and/or distant metastases received only palliative treatment, and were omitted from the survival analyses.

### Statistical analysis

Statistical analyses were carried out using the SAS System for Windows, release 9.4 (SAS Institute Inc., Cary, NC). Mean ages in the TLR groups were compared with 1-way analysis of variance using the Tukey’s method for pairwise comparisons. The associations of categorical variables with different TLR statuses were compared with Pearson chi-squared test or Fisher’s exact test. Survival rates were calculated using the Kaplan-Meier method. Follow-up time was calculated from the end of the primary treatment, usually from the last day of radiotherapy, to the end of follow up or the date of death. Log-rank test was used to compare Kaplan-Meier survival curves. Age-adjusted and multivariable-adjusted Cox regression was used to test the association of TLR status with disease-specific survival (DSS) and overall survival (OS). The multivariable Cox regression analysis was adjusted for the potential confounding factors age, gender, stage and viral status. To avoid multicollinearity problems histology was excluded from the multivariable model due to the high correlation with viral status. The results are expressed using hazard ratios (HRs) with 95% confidence intervals (CIs). *P*-values of less than 0.05 were considered as statistically significant.

## Results

### Overall characterization of the patients

We analyzed the clinical data of 150 patients who had appropriate tissue specimens in TMA (Table 2). The mean age (SD) of the patients was 57.0 [15] years, and 101/150 (67%) were men. Almost all (145/150, 97%) patients were Caucasians of Finnish ethnic background. The majority (63%) presented with stage III/IV disease at the time of the diagnosis. Thirty-three patients (22%) had KSCC, 25 patients (17%) had non-keratinizing differentiated carcinoma (NK-D), and 92 patients (61%) had non-keratinizing undifferentiated carcinoma (NK-U). There were no basaloid SCCs. More detailed information on the patients has been reported in our earlier study [13].

**Table 2:** Patient characteristics

Characteristic	n (%)
All patients	150 (100)
Gender	
Male	101 (67)
Female	49 (33)
Mean age at diagnosis	
Years (SD)	57.0 (15)
Range	12–85
Ethnicity	
Finnish	145 (97)
Other^a^	5 (3)
Smoking	
Smoker or ex-smoker	71 (47)
Non-smoker	36 (24)
Not known	43 (29)
T class	
T1	54 (36)
T2	40 (27)
T3	26 (17)
T4	30 (20)
N class	
N0	53 (35)
N1	33 (22)
N2	51 (34)
N3a	10 (7)
N3b	3 (2)
Overall stage	
I	19 (12)
II	37 (25)
III	52 (35)
IV	42 (28)
Histology	
Keratinizing squamous cell carcinoma	33 (22)
Non-keratinizing differentiated carcinoma	25 (17)
Non-keratinizing undifferentiated carcinoma	92 (61)
Virus status	
EBV-positive	93 (62)
HPV-positive	21 (14)
EBV/HPV-negative	36 (24)
Treatment	
Radiotherapy	67 (45)
Chemoradiotherapy	76 (51)
Palliative	7 (4)
Irradiation technique	
2-dimensional radiotherapy (2D)	15 (10)
3-dimensional radiotherapy (3D)	88 (60)
Intensity modulated radiotherapy (IMRT)	44 (30)

^a^ Three from South-East Asia, one from Africa, one from Eastern Europe

### EBV and HPV status

Of the total of 150 tumours, 93 (62%) were positive in EBER ISH and thus regarded as EBV-positive. HPV positivity was examined with p16 immunohistochemistry and high risk HPV E6/E7 mRNA ISH, and 21/150 (14%) tumours were positive with both methods forming the HPV-positive group. It is worthy to note that all p16-positive tumours showed positivity in HPV mRNA ISH; the concordance between these two methods was 100%. There were no co-infections with EBV and HPV. In 36/150 (24%) cases the tumours were negative for both EBV and HPV (EBV/HPV-negative group) [13].

### Overall TLR expression in NPC and in benign adenoid tissues

TLR1, TLR2, TLR4, TLR7, and TLR9 were very highly expressed in NPC (Table 3). In TLR5 staining only, the proportion of negative samples was as high as 39%. TLR1 was found in the cytoplasm of cancer cells in 146/149 (98%) samples, and TLR1 positivity was strong in 82% of all samples. TLR2 was expressed in the cytoplasm and in the nuclei in 133/141 (94%) cases. In most of the samples (66%), nuclear staining was similar or milder than cytoplasmic staining, while in 28% of the samples it was stronger. TLR4 was positive in the cytoplasm of 130/141 (92%) specimens, and the percentages for mild and strong expression were 35 and 57%, respectively. Also in TLR5 staining, intensities in nuclear membranous and cytoplasmic staining were divisible into mild (53/143, 37%) and strong (32/143, 22%). TLR7 expression was found on the nuclear membranes and partly in the nuclei in 134/143 (94%) samples. Occasional large cancer cells also had cytoplasmic TLR7 staining. Mild TLR7 expression was found in 45/143 (31%) and moderate or strong expression in 89/143 (62%) cases. We united the groups with moderate and strong TLR7 expression, as these categories gave very similar results. TLR9 was expressed in the cytoplasm of 145/149 (97%) samples. Five controls of benign adenoid tissues expressed TLR1 and TLR2 in similar locations as in NPC tissues (Table 1). However, TLR4, TLR5, TLR7, and TLR9 were expressed differently in benign samples. TLR4 expression was also found in the nuclei of the benign cells. TLR5 was expressed on the nuclear membranes and in the cytoplasm in both malignant NPC and benign adenoid tissues, but the benign nasopharyngeal epithelium expressed TLR5 in the basal cells only (Fig. 1g), while the expression was ubiquitous in NPC (Fig. 1c, d). In benign adenoid tissues, TLR7 was expressed in the cytoplasm, while the nuclear membranes, positive in NPC, were not stained (Fig. 1h). TLR9 was found both in the cytoplasm and on the plasma membranes in the adenoid samples. The scorings were repeated with blinding of the data, and similar results were obtained.

**Table 3:** Expression of different TLRs in NPC in Finland

TLR type	Intensity of TLR expression, n (%)			
	Negative	Mild	Strong	Total
TLR1	3 (2)	24 (16)	122 (82)	149
TLR2^nucl^	8 (6)	93 (66)	40 (28)	141
TLR2^cyto^	8 (6)	59 (42)	74 (52)	141
TLR4	11 (8)	50 (35)	80 (57)	141
TLR5	58 (39)	53 (37)	32 (22)	143
TLR7	9 (7)	45 (31)	89 (62)	143
TLR9	4 (3)	61 (41)	84 (56)	149

### Association of TLR expression with patient characteristics and tumour histology

TLR2 and TLR5 expression associated with age. The patients with mild TLR2^nucl^ expression were significantly younger than the patients with strong TLR2^nucl^ expression with mean (SD) ages of 54.7 [16] and 61.9 [13], respectively (*p* = 0.036). In addition, the patients with negative or mild TLR5 expression were younger than those with strong TLR5 expression with mean ages of 54.4 [15], 55.5 [16], and 63.4 [13], respectively (*p* = 0.021 and *p* = 0.053). There were significantly more women in the group with strong TLR5 expression (50%) compared to the group with negative TLR5 expression (24%, *p* = 0.013). Furthermore, TLR2^nucl^ and TLR5 expressions correlated with histology (*p* = 0.006 and *p* < 0.0001, respectively): keratinizing tumours had often strong TLR2^nucl^ (18/31, 58%) and strong TLR5 (19/32, 59%) expressions, while undifferentiated tumours presented mostly with mild TLR2^nucl^ (65/87, 75%) and negative TLR5 (48/88, 55%) expressions. No statistically significant associations were found between any TLR and smoking, TNM classification, or overall stage.

### Association between TLR expression and viral status

**Fig. 2:**
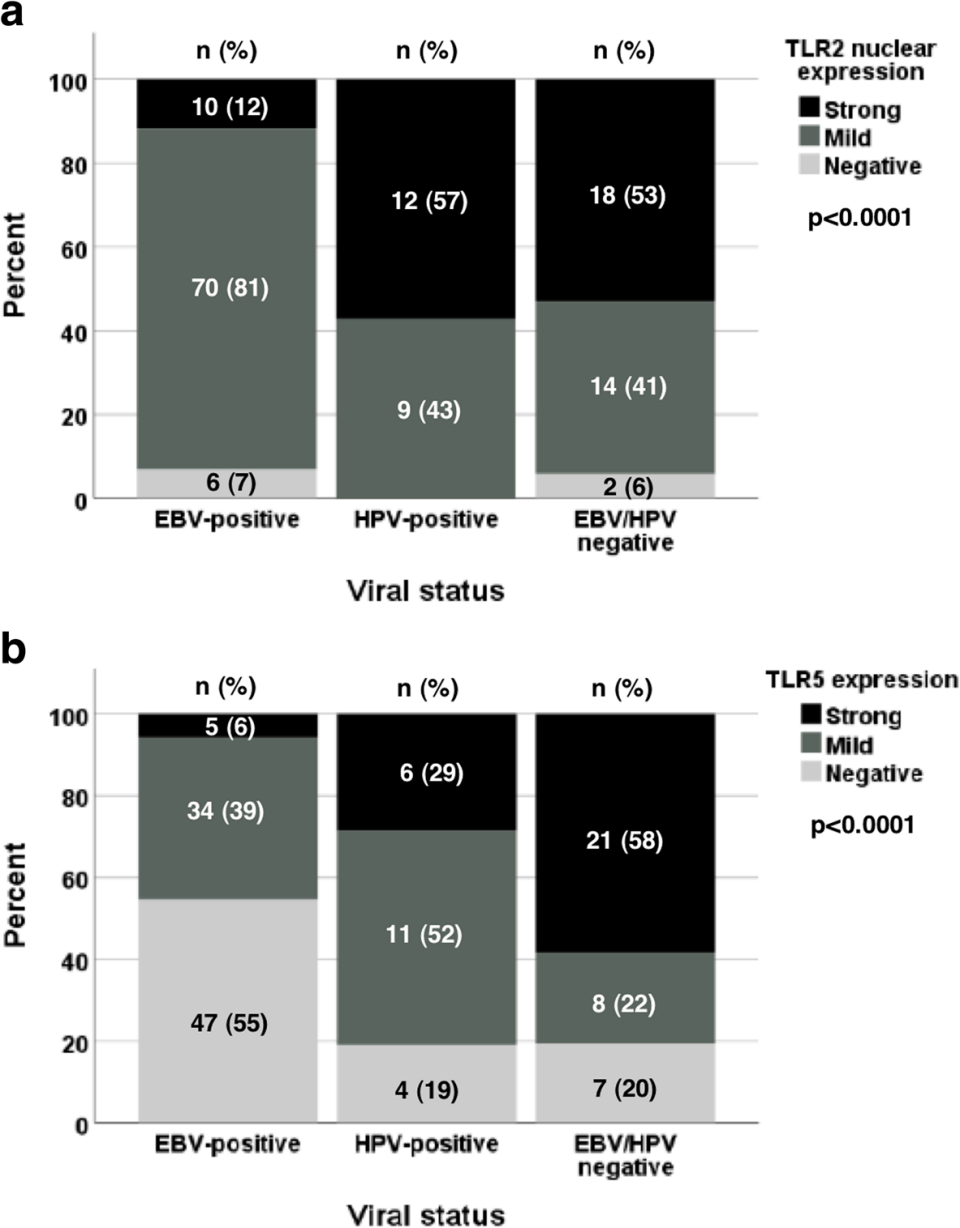
Score distribution of TLR expressions according to viral status. **a** TLR2^nucl^. **b** TLR5

Cases were classified into three groups according to their viral status as follows: EBV-positive, HPV-positive, and EBV/HPV-negative. We found that TLR1, TLR4, TLR7, and TLR9 expressions were not related to viral status, while the opposite was true for the expressions of TLR2 and TLR5 (p < 0.0001 in both). The expression of TLR2^nucl^ was stronger in the HPV-positive group and in the EBV/HPV-negative group than in the EBV-positive group. Accordingly, strong TLR2^nucl^ positivity was seen in 57 and 53% versus 12% of the cases in each viral status group, respectively. The same tendency was seen in TLR5 expression, which was strong in 58% of the EBV/HPV-negative tumours, strong (29%) or mild (52%) in the HPV-positive tumours, and negative in 55% of the EBV-positive cases. Figure 2 shows the relationship between viral status and TLR2^nucl^/TLR5 expressions.

### Association of TLR expression with clinical outcome

**Fig. 3:**
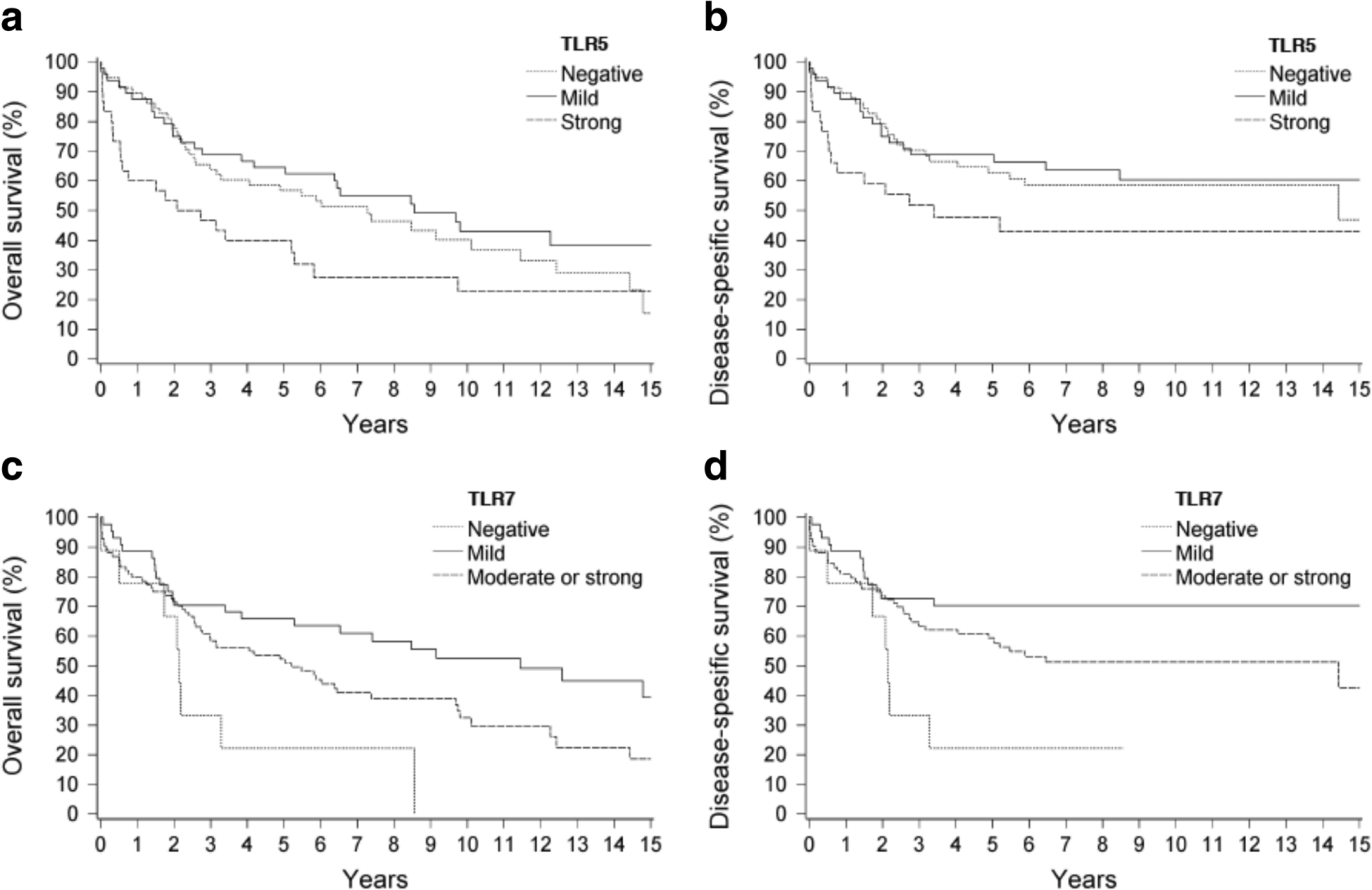
Kaplan-Meier survival curves. **a** Overall survival for TLR5. **b** Disease-specific survival for TLR5. **c** Overall survival for TLR7. **d** Disease-specific survival for TLR7

The median follow-up time was 63 months for patients treated with curative intent (*n* = 143) and for most patients (97%) it was 5 years or more. In Kaplan-Meier analysis, the patients with strong TLR5 expression had worse OS than those with mild or negative TLR5 expression (Fig. 3a; *p* = 0.019). The 5-year OS rates according to the TLR5 expression categories were 65% (mild), 57% (negative), and 40% (strong). DSS was nearly significantly worse in the patients with strong TLR5 expression compared to the patients with mild or negative expression (Fig. 3b; *p* = 0.062). The 5-year DSS rates were 69% (mild), 63% (negative), and 48% (strong). In contrast, the patients with no TLR7 expression had worse OS and DSS than the patients with positive TLR7 staining (Fig. 3c and d; *p* = 0.008 and p = 0.019, respectively). The 5-year OS rates according to TLR7 expression were 66% (mild), 52% (moderate or strong), and 22% (negative), and the corresponding DSS rates were 70% (mild), 59% (moderate or strong), and 22% (negative). The patients with negative TLR9 expression had worse OS compared to the patients with positive TLR9 expression, but the significance of this is limited by the presence of only four TLR9-negative cases. The 5-year OS rates according to TLR9 expression were 63% (mild), 52% (strong), and 25% (negative). In Kaplan-Meier analysis, we did not find survival differences related to TLR1, TLR2, and TLR4 expression.

### TLR7 and TLR9 as prognostic factors in NPC

In multivariable-adjusted Cox regression analysis, positive TLR7 expression (mild, *p* = 0.018; moderate or strong, *p* = 0.038) compared to negative TLR7 expression was a significant prognostic factor for a better OS (Table 4). When analyzing DSS in multivariable-adjusted Cox regression analysis, only mild TLR7 expression (*p* = 0.046) compared to negative TLR7 expression remained as a significant prognostic factor for a better DSS. In age-adjusted Cox regression analysis, positive TLR9 expression (mild, *p* = 0.004; strong, *p* = 0.034) compared to no expression was associated with a better OS. However, when adjusted for gender, stage, and viral status TLR9 expression did not remain as a significant prognostic factor.

**Table 4:** Age-adjusted and multivariable Cox regression analysis of 143 patients for overall survival (a) and disease-specific survival (b)

	Age-adjusted		Multivariable^a^	
	HR (95% CI)	*p*-value	HR (95% CI)	*p*-value
a) Overall survival (OS)				
Stage				
II vs. I	2.13 (0.96–4.73)	0.064 NS		
III vs. I	2.79 (1.30–5.95)	0.008		
IV vs. I	4.52 (2.12–9.64)	< 0.001		
Viral status				
HPV-pos vs. EBV-pos	1.62 (0.88–3.00)	0.123 NS		
HPV/EBV-neg vs. EBV-pos	2.67 (1.64–4.34)	< 0.001		
TLR1				
Mild vs. neg	0.30 (0.09–1.04)	0.057 NS	0.69 (0.18–2.70)	0.595 NS
Strong vs. neg	0.24 (0.08–0.79)	0.018	0.48 (0.14–1.70)	0.256 NS
TLR2 nuclear				
Mild vs. neg	0.56 (0.25–1.24)	0.151 NS	0.53 (0.23–1.19)	0.125 NS
Strong vs. neg	0.62 (0.27–1.45)	0.271 NS	0.52 (0.21–1.30)	0.162 NS
TLR2 cytoplasmic				
Mild vs. neg	0.62 (0.27–1.41)	0.256 NS	0.49 (0.20–1.17)	0.109 NS
Strong vs. neg	0.70 (0.31–1.55)	0.373 NS	0.68 (0.30–1.56)	0.363 NS
TLR4				
Mild vs. neg	1.16 (0.51–2.65)	0.727 NS	1.07 (0.46–2.50)	0.872 NS
Moderate/strong vs. neg	1.15 (0.52–2.55)	0.731 NS	1.13 (0.50–2.56)	0.775 NS
TLR5				
Mild vs. neg	0.75 (0.45–1.24)	0.259 NS	0.65 (0.38–1.10)	0.108 NS
Strong vs. neg	1.37 (0.80–2.33)	0.250 NS	0.88 (0.47–1.61)	0.666 NS
TLR7				
Mild vs. neg	0.28 (0.12–0.64)	0.003	0.37 (0.16–0.84)	0.018
Moderate/strong vs. neg	0.49 (0.23–1.04)	0.062 NS	0.44 (0.20–0.95)	0.038
TLR9				
Mild vs. neg	0.21 (0.07–0.60)	0.004	0.37 (0.12–1.16)	0.087 NS
Strong vs. neg	0.32 (0.12–0.92)	0.034	0.49 (0.17–1.46)	0.199 NS
b) Disease-specific survival (DSS)				
Stage				
II vs. I	2.50 (0.68–9.14)	0.166 NS		
III vs. I	4.72 (1.40–15.95)	0.013		
IV vs. I	8.63 (2.59–28.80)	< 0.001		
Viral status				
HPV-pos vs. EBV-pos	1.10 (0.49–2.47)	0.822 NS		
HPV/EBV-neg vs. EBV-pos	2.22 (1.25–3.96)	0.007		
TLR1				
Mild vs. neg	0.50 (0.11–2.25)	0.369 NS	1.30 (0.26–6.57)	0.748 NS
Strong vs. neg	0.32 (0.08–1.32)	0.113 NS	0.76 (0.17–3.43)	0.720 NS
TLR2 nuclear				
Mild vs. neg	0.75 (0.26–2.10)	0.578 NS	0.88 (0.31–2.52)	0.811 NS
Strong vs. neg	0.84 (0.28–2.54)	0.757 NS	1.05 (0.33–3.34)	0.937 NS
TLR2 cytoplasmic				
Mild vs. neg	0.62 (0.23–1.65)	0.336 NS	0.69 (0.25–1.90)	0.468 NS
Strong vs. neg	0.71 (0.28–1.84)	0.481 NS	0.89 (0.33–2.34)	0.806 NS
TLR4				
Mild vs. neg	1.52 (0.54–4.41)	0.441 NS	1.65 (0.56–4.88)	0.368 NS
Moderate/strong vs. neg	1.12 (0.40–3.20)	0.827 NS	1.28 (0.44–3.73)	0.652 NS
TLR5				
Mild vs. neg	0.87 (0.47–1.61)	0.665 NS	0.81 (0.43–1.53)	0.518 NS
Strong vs. neg	1.62 (0.85–3.06)	0.141 NS	1.34 (0.64–2.80)	0.442 NS
TLR7				
Mild vs. neg	0.28 (0.11–0.70)	0.006	0.39 (0.15–0.98)	0.046
Moderate/strong vs. neg	0.47 (0.21–1.05)	0.067 NS	0.47 (0.21–1.09)	0.079 NS
TLR9				
Mild vs. neg	0.27 (0.08–0.93)	0.038	0.54 (0.15–1.98)	0.351 NS
Strong vs. neg	0.39 (0.12–1.29)	0.123 NS	0.70 (0.20–2.43)	0.576 NS

^a^ Adjusted for age, gender, stage, and viral status

## Discussion

TLR signalling has been associated with tumour development [25]. The expression pattern and the function of TLRs in tumour progression seem to be cell-type specific and relate to different conditions, such as infections [5]. In our whole population-based study of NPC in Finland, we examined the expression of TLR1, TLR2, TLR4, TLR5, TLR7, and TLR9 in 150 tumours and analysed their pattern of expression according to EBV and HPV status, and clinical outcome. To our knowledge, this is the first study on TLRs in non-endemic NPC published to date. A few studies, conducted in high-incidence areas, have reported that genetic polymorphisms of TLR3, TLR4, TLR9, and TLR10 are associated with a risk of developing NPC in endemic populations [19–22], but outcome results related to TLR expression are lacking worldwide.

The present study demonstrated that the expression patterns of TLR2 and TLR5 were related to the viral status while both TLRs were expressed significantly less in EBV-positive than in HPV-positive or EBV/HPV-negative NPC. These expressions were also related to well-established prognostic factors such as age and histology [23], which tend to dilute the independent prognostic significance of TLR2 and TLR5 in multivariable Cox regression analysis. However, in Kaplan-Meier analysis, the patients with strong TLR5 expression had worse survival compared to those with mild or negative expression (Fig. 3). This is in line with clinical studies on OPSCC and oral tongue squamous cell carcinoma (OTSCC), which report the association of poor DSS with strong expression of TLR5 [17, 26]. The exact effects of TLR-mediation on tumour growth are not known, but several in vitro studies on other types of carcinomas have shown that activation of TLR5 can promote tumorigenesis. For example, such activation has enhanced the proliferation of gastric cancer cells, and the migration and invasion of salivary gland adenocarcinoma [27, 28].

In contrast to TLR5, the patients with positive TLR7 expression had better DSS and OS than the patients with no TLR7 expression, and TLR7 was found to be a significant prognostic factor in multivariable Cox regression analysis. The finding that the patients with mild TLR7 expression had slightly better 5-year survival than the patients with strong TLR7 expression was not expected, but a similar result has been reported in patients with oral squamous cell carcinoma [29]. However, that report did not mention the survival rates of the patients with TR7-negative tumours. Nevertheless, it described variable TLR7 expression patterns when comparing between normal, dysplastic, and carcinoma tissues [29]. In our study, TLR7 expression in NPC samples was prominent on nuclear membranes, while in benign tissues TLR7 was expressed in the cytoplasm and in the nuclei. Another example of a defensive marker with derangement of function, in both over- and underexpression, is matrix metalloproteinase-8 [30].

Our findings suggest that viruses, or tumours related to specific viruses, or simply malignant neoplasms evoke distinct immunological reactions, but the hosts’ responses vary because of yet unknown causes. This might partly explain the differences in the effectiveness of oncological treatments. However, according to the hypothesis proposed by Wee et al. [31], causation may also be the reverse. They suggested that genetic TLR polymorphisms, especially in X-chromosome-linked TLR8, affect the innate immune response and make certain populations and individuals more vulnerable to infection-related cancers [31]. In NPC, altered TLR function could allow the virus to enter the nasopharyngeal mucosa and cause persistent infection finally resulting in a carcinogenic process [31]. We did not evaluate TLR8 expression in our tumour cells, but we found the usual but unexplained male preponderance in EBV- and HPV-positive patients (74 and 76% were males, respectively). In line with this, there were significantly more women in the virus-negative group (56%) compared to the EBV-positive (*p* = 0.001) and the HPV-positive (*p* = 0.020) groups. This suggests that men are more susceptible to virus-related NPC than women even in a non-endemic low-incidence population.

We studied TLR expression by immunohistochemistry to determine the expression sites of TLRs in carcinoma cells. As stated in a recent review article by Hamonic et al. [5], comprehensive understanding of the changing localizations of TLRs could aid us in understanding the basis of cancer immunology and possibly in developing new treatment modalities. We found that both in the benign adenoid control tissues and in the NPC samples, TLR1, TLR2, TLR4, and TLR5 were expressed diffusely in the cytoplasm instead of the cell surface, where they have been reported to localize when activated by pathogen-associated molecular patterns [32]. Interestingly, TLR4 expression was detected in the nuclei of benign controls but not in the nuclei of NPC cells. In benign samples, TLR5 was expressed exclusively in the basal layer of the nasopharyngeal epithelium as reported also for the normal oral mucosa [33], while in NPC the expression was diffuse. Similar to NPC, Pimentel-Nunes et al. have reported changing TLR localizations in gastric carcinogenesis [34]. They found that the normal gastric mucosa expressed TLR2, TLR4, and TLR5 in a polarized manner in the apical and particularly the basolateral membrane. By contrast, in metaplasia, dysplasia, and adenocarcinoma, these TLRs were expressed throughout the cytoplasm with no apparent polarization [34]. Also in previous studies on OPSCC and OTSCC, TLR5 has been localized in the cytoplasm rather than the membranes of the neoplastic cells [26, 35]. These findings suggest that activation of TLRs in abnormal locations may be related to carcinogenetic processes. We can only speculate whether TLRs have different functions in benign and malignant tissues, or if they become activated in different cellular compartments by PAMPs or DAMPs. Further research is needed to describe the mechanisms and causal connections between these phenomena.

In the present study, the retrospective nature of patient data carries limitations regarding the history of smoking habits and alcohol consumption. This limited our possibilities to evaluate the impact of these known carcinogens on the differences in expression of the studied TLRs. However, the uniform nationwide health care system in Finland enabled us to collect complete treatment and follow-up data, and a high proportion of diagnostic histopathological samples for TMA. In fact, histopathology was available for 150 (72%) of a total of 207 patients. While the size of the Finnish population limited the number of cases, we can nevertheless attest that the present cases represent a truly whole population-based material for the NPC patients treated during this 20-year period [36].

## Conclusions

Our study is the first to evaluate TLR expressions in NPC of a non-endemic area. We found that TLRs were highly expressed in NPC, and intensity of TLR2 and TLR5 expressions were related to viral status. Expressions of TLR2 and TLR5 were strong in virus-negative cases, which will logically lead us to further studies on other possible factors causing NPC. In addition, TLR7 seems to be an independent prognostic factor of non-endemic NPC.

## Data Availability

TMAs can be found in Auria Biobank, Turku University Hospital. Please contact the corresponding author for data enquiries.

## Abbreviations

CI: Confidence interval
DAB: Diaminobenzidine
DAMP: Damage-associated molecular pattern
DSS: Disease-specific survival
EBER: EBV-encoded RNA
EBV: Epstein-Barr virus
EDTA: Ethylenediaminetetra-acetic acid
FFPE: Formalin-fixed paraffin-embedded
Gy: Gray
HPV: Human papillomavirus
HR: Hazard ratio
ISH: In situ hybridization
KSCC: Keratinizing squamous cell carcinoma
NK-D: Non-keratinizing differentiated carcinoma
NK-U: Non-keratinizing undifferentiated carcinoma
NPC: Nasopharyngeal carcinoma
OPSCC: Oropharyngeal squamous cell carcinoma
OS: Overall survival
OTSCC: Oral tongue squamous cell carcinoma
PAMP: Pathogen-associated molecular pattern
TLR: Toll-like receptor
TMA: Tissue microarray
UICC: International union against cancer
